# Assessment of infectivity and the impact on neutralizing activity of immune sera of the COVID-19 variant, CAL.20C

**DOI:** 10.1038/s41392-021-00695-0

**Published:** 2021-07-27

**Authors:** Zhongcheng Zhou, Peng Du, Meixing Yu, Daniel T. Baptista-Hon, Man Miao, Andy P. Xiang, Johnson Yiu-Nam Lau, COVID-19 Immunity Investigation Group, Ning Li, Xinxin Xiong, Hong Huang, Zhihai Liu, Qinjin Dai, Jie Zhu, Shanyun Wu, Gen Li, Kang Zhang

**Affiliations:** 1 Guangzhou Women and Children’s Medical Center, Guangzhou Medical University, Guangzhou, China; 2 University Hospital and Center for Biomedicine and Innovations, Faculty of Medicine, Macau University of Science and Technology, Macau, China; 3 Center for Stem Cell Biology and Tissue Engineering, Key Laboratory for Stem Cells and Tissue Engineering, Ministry of Education, Sun Yat-sen University, Guangzhou, China; 4 Department of Biology, Hong Kong Baptist University, Kowloon Tong, Hong Kong China; 5 Department of Bioinformatics and AI, Bioland Laboratory, Guangzhou, China; 6 Guangzhou Women and Children’s Medical Center, Guangzhou Medical University, Guangzhou, China

**Keywords:** Infectious diseases

**Dear Editor**,

New variants of SARS-CoV-2 have been emerging since the initial outbreak in 2019;^1^ one of the latest ones was identified in Southern California in October 2020 and was subsequently detected in 26 other states in the United States as well as other countries as of January 2021. This strain, derived from coronavirus D614G mutation, is characterized by three genetic variations leading to three novel amino acid substitutions (S13I, W152C, and L452R) in the spike (S) protein (Supplementary Fig. S1). The S13I and W152C are in the N-terminal domain, and more importantly, the L452R is located in the receptor-binding domain (RBD) (Supplementary Fig. S3).

To determine how these nonsynonymous substitutions in CAL.20C strain in the background of D614G of SARS-CoV-2 S protein affect infectivity and whether these amino acid substitutions can compromise the neutralizing immune responses from nonhuman primates vaccinated from an RBD protein vaccine,^2^ patients who recovered from COVID-19 infection and recipients who were vaccinated with Pfizer-BioNTech mRNA, we constructed various lentivirus-based pseudotyped viruses that expressed different variants of the S protein (containing different combinations of amino acid substitutions, Supplementary Fig. S1).

Our data showed that D614G is associated with a significant increase (4.6×) in infectivity compared to the wild type in 293T-ACE2-TMPRSS2 (which express human ACE2 and TMPRSS2, Supplementary Fig. S2) cells and this is in accordance with the results from previous studies^3^ (Fig. 1a). Interestingly, CAL.20C showed a little higher infectivity (but no statistical significance) compared with the wild type, but lower than the D614G variant. When individual amino acid substitutions were evaluated in the D614 background, the L452R-D614G variant pseudovirus displayed 2.4-fold increase in infectivity compared to the D614G, while the W152C-D614G variant showed similar infectivity as D614G and the S13I-D614G variant exhibited lower infectivity (Fig. 1a). More interestingly, all three variants, S13I-D614G, W152C-D614G, and L452R-D614G, showed higher infectivity compared to CAL.20C (Fig. 1a), suggesting that CAL.20C did not evolve naturally based on infectivity alone and other biologic fitness factors were involved in the evolution of this variant.

**Fig. 1:**
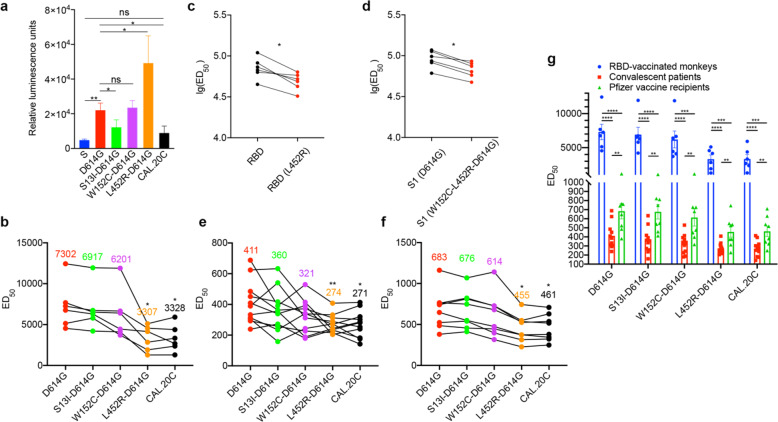
Infectivity and neutralizing activities of immune sera for CAL.20C variants. **a** Infectivity levels with a pseudotyped virus. *X*-axis shows different COVID-19 S variants and *Y*-axis shows relative luminescence unit determined by infectivity assay (compared to the cell culture control). **b** Comparison between the binding affinity of RBD and RBD (L452R) with immune sera from nonhuman primates by ELISA (*n* = 6). **c** Comparison between the binding affinity of S1 (D614G) and S1 (W152C-L452R-D614G) with immune sera from nonhuman primates by ELISA (*n* = 6). **d** Neutralization activities of the immune sera from nonhuman primates vaccinated with an RBD protein vaccine (*n* = 6). **e** Neutralization activities of sera from COVID-19 infection convalescent patients against various pseudotyped viruses expressing COVID-19 S variants related to CAL.20C (*n* = 11). **f** Neutralization activities of sera from vaccine recipients (*n* = 8, Pfizer-BioNTech) against various pseudotyped viruses expressing COVID-19 S variants related to CAL.20C. **g** Cross-comparison of neutralization activities of sera from the RBD-vaccinated monkeys (*n* = 6), COVID-19 convalescent patients (*n* = 11), and mRNA vaccine recipients (*n* = 8, Pfizer-BioNTech) against various pseudotyped viruses expressing COVID-19 S variants related to CAL.20C. In **b**–**g**, *X*-axis shows different COVID-19 S variants and *Y*-axis shows ED_50_ or lg(ED_50_), values of serial dilution for 50% effective neutralization. n.s. nonsignificant. *P < 0.05, **P < 0.01, ***P < 0.001, and ****P < 0.0001

For vaccinated monkey sera, when compared with D614G pseudovirus, both CAL.20C and L452R-D614G pseudotyped viruses showed markedly reduced neutralization with a median of 2.4- and 2.3-fold, respectively (Fig. 1b). There were no significant differences in neutralization between D614G and S13I-D614G or W152C-D614G (Fig. 1b). To further characterize the role of L452R in the binding of neutralizing antibodies to the S protein variants, the binding affinities of the various protein constructs were tested by enzyme-linked immunosorbent assay (ELISA). Figure 1c showed the relatively higher binding activity to RBD vs RBD(L452R), suggesting a consistent drop of binding of the L452R (*n* = 6). Figure 1d showed the impact of binding activity of the W152C-L452R-D614G S1 protein compared to D614G (*n* = 6).

Next, we determined the effects of CAL.20C and its related pseudotyped viruses on the viral neutralizing activities in sera from patients recovered from COVID-19 infection (*n* = 11) and from vaccine recipients (Pfizer-BioNTech) (*n* = 8). For convalescent sera, a significant loss of neutralization activity was observed against CAL.20C and the L452R-D614G when compared to D614G, with a median reduction of 1.5-fold for both comparisons (Fig. 1e). There was some minor reduction but a statistically insignificant reduction for W152C-D614G, and no difference was observed between S13I-D614G and D614G pseudotyped viruses when compared to D614G (Fig. 1e). For vaccine sera, there was also a significant reduction of neutralization activity against CAL.20C and L452R-D614G compared to D614G, with a median reduction of about 1.5-fold for both comparisons (Fig. 1f). From a cross-comparison of neutralization activities of sera from the RBD-vaccinated monkeys, COVID-19 convalescent patients, and mRNA vaccine recipients, RBD-vaccinated monkey’s sera exhibited the best protective effects, and the mRNA vaccine showed a better protective effect than the COVID-19 convalescent sera in all S variants (Fig. 1g).

Our data showed a few important points. First, the naturally evolved COVID-19 variant CAL.20C has a little higher infectivity than the initially prevalent S^D614^ virus, but much lower than the recently evolved D614G variant.^3^ Second, among the three amino acid substitutions, L452R, which is an amino acid located on the RBD, was associated with high infectivity in the L452R-D614G format. Given its location within RBD, L452R was also associated with a reduction in the neutralizing activity of sera from the nonhuman vaccinated primates, convalescent patients subjects, and vaccine recipients. This observation could be explained at least in part by a reduction of the binding of the L452R-D614G to the neutralizing sera by ELISA. Third, interestingly, although L452R was found to have a major impact on infectivity, the addition of the other two amino acid substitutions S13I-W152C reduced the infectivity of the L452R, rendering the resultant CAL.20C less infective compared to the D614 backbone. If the pseudotyped virus assay truly represents the real viral infection, then the infectivity is not the major driver for the natural evolution of the CAL.20C variant. The S13I and W152C may confer other replicative, structural, and host–virus interaction advantages that facilitate their evolution. In addition, S13I and W152C did not have any major impact on the neutralizing activity of the immune sera from nonhuman vaccinated primates, convalescent patient subjects, and vaccine recipients.

A recent work also reported data consistent with our findings reported here.^4^ Their study used the B.1.427/B.1.429 viral isolates for the neutralization assay and here we have both single and combined B.1.427/B.1.429 pseudotyped viruses for neutralization. However, Garcia-Beltran et al. reported no difference in the neutralization between B.1.427/B.1.429 and wild-type or D614G lenti-SARS-CoV-2 S pseudovirus.^5^ Certainly, this issue should be addressed by more laboratories using different systems so that we can have a clearer picture of the implications of these amino acid substitutions generated through natural evolution.

According to our results, the CAL.20C mutations do reduce but not totally escape the neutralizing activity of the immune sera so far. The current vaccines based on the original RBD sequence and synthetic spike mRNA are still able to provide some degrees of protective immunity against infection by the newly emerged CAL.20C strains. Several independent L452R-carrying lineages have recently emerged across the globe, and the B.1.617 or “double mutant” Indian variant (carrying L452R) is playing in the world’s fastest-growing surge of COVID-19 cases in India. Close monitoring of these variants containing L452R may guide us to the need of developing an additional COVID-19 vaccine with L452R in the RBD. The same implication can be applied to the development of therapeutic neutralizing antibodies.

## Supplementary information

Supplementory

## Data Availability

Data are available upon reasonable request.
