# Supplementary material for: Assessment of infectivity and the impact on neutralizing activity of immune sera of the COVID-19 variant, CAL.20C
Source: Signal Transduct Target Ther. 2021 Jul 27;6:285. doi: 10.1038/s41392-021-00695-0 (PMC8313410; doi:10.1038/s41392-021-00695-0)
Supplement: Supplementary file 1 — Supplementory [file 41392_2021_695_MOESM1_ESM.docx]

**Supplementary Materials for**

**Assessment of Infectivity and the Impact on Neutralizing Activity of Immune Sera of the COVID-19 Variant, CAL.20C**

Zhongcheng Zhou, Peng Du, Meixing Yu, Daniel T. Baptista-Hon, Man Miao, Andy P. Xiang, Johnson Yiu-Nam Lau, COVID-19 Immunity Investigation Group, Gen Li, Kang Zhang

Editorial correspondence:

Kang Zhang, MD, PhD, email address: kang.zhang@gmail.com

This file includes:

Materials and Methods

Figure. S1 to S3

Table S1 to S2

**Materials and Methods**

**Construction of the COVID-19 variant S protein expression plasmids**

The codon optimized full-length S protein gene was synthesized and cloned into the pCAGGS vector (Genscript, Nanjing, China). A site-directed mutagenesis approach was employed to generate D614G, S13I, W152C, L452R and CAL.20C mutants, with primers listed in Supplementary Table 1. The PCR Mix and recombinant enzyme used were high-fidelity DNA polymerase Mix (P525, Vazyme) and Exnase II (C214, Vazyme). All plasmid sequences were confirmed by Sanger’s method.

**Human subjects**

The study was approved by the Medical Ethics Committee of the Guangzhou Women and Children’s Hospital. The COVID-19 patients enrolled were diagnosed between January and March 2020 and were managed at the designated hospitals in Wuhan and surrounding cities in the Hubei Province^1,2^, and all were confirmed to have COVID-19 by positive RT-PCR testing(s). The demographic and clinical information was previously reported^1,2^. Sera from vaccine recipients were obtained from volunteers who were vaccinated by an mRNA vaccine made by BioNTech/Pfizer in the University Hospital after obtaining an informed consent at Macao University of Science and Technology.

**Sera from RBD-immunized non-human primates**

All procedures involved in the non-human primate study were reviewed and approved by the Institutional Animal Care and Use Committee of Institute of Sun Yat-sen University. Five adult non-human primates *Cynomolgus macaques (Macaca fascicularis)* (5-9 years old) were employed for the vaccine study, which were immunized with 20 μg RBD protein with Al(OH)_3_ plus topical imiquimod (n=6). The detailed methods for vaccination, booster administration and other experimental details are provided in our recent report^3^.

**Production and quantification of pseudotyped virus particles**

5×10^6^ 293T cells in 100 mm dish were co-transfected with 12 μg pLOVE-luciferase-EGFP plasmid, 6 μg psPAX2 and 2 μg S or S variants plasmids using Lipofectamine 3000 (Invitrogen, L30000015) according to the manufacturer’s instruction. The medium for transfected cells were replaced by 10 ml of fresh medium after 6~8 hours, and the supernatant containing SARS-CoV-2 pseudotyped viruses were harvested and filtered through 0.45 μm filter 48 hours after transfection. RNA of 100 μl SARS-CoV-2 pseudotyped virus and the related constructed pseudotyped viruses were extracted using the MiniBEST Viral RNA/DNA Extraction Kit Ver.5.0 (TaKaRa, 9766). The virus DNA was obtained by reverse transcription using the HiScript® III All-in-one RT SuperMix Perfect for qPCR (Vazyme). RT-PCR was performed using TransLv Lentivirus qPCR Titration Kit (TransGen, FV201).

**Infectivity assay**

The 293T-ACE2-TMPRSS2 cells (1×10^4^/100 μl/well) were seeded in 96-well plates. After quantification by RT-PCR, the pseudotyped viruses were diluted to 80,000 particle number in 100 μl DMEM medium, and 100 μl of the virus suspension was added per well into the 96-well cell culture plates. After 12 hours of infection, the medium was replaced with 10 ml fresh culture medium to each well. Luciferase activity was assayed after another 48 h of incubation in a tissue culture chamber (5% CO_2_ at 37°C). Luciferase substrate was mixed with cell lysis buffer (Promega, E6120) and was added to the plate (100 μl/well). After two minutes, 100 μl of lysate was transferred to an opaque 96-well plate and the luminescence signal was detected using TECAN Infinite P500.

**Neutralization assay**

The effects of the sera and monoclonal antibodies on entry inhibition by the pseudotyped viruses were evaluated through the measurement of a reduction of the luciferase gene expression. The 293T-ACE2-TMPRSS2 cells (1×10^4^/100μl/well) were seeded in 96-well plates. The samples were serially diluted two times (50-folds as the initial dilution) for a total of eight gradients in 96 well plates. The virus solution was subsequently added to the wells. Six virus control wells (without antibody samples) and six control wells (cells without virus or antibody samples) were also included for each 96-well plate. The 96-well plates were incubated at 37°C for 1 hour. After incubating in tissue incubator (5% CO_2_ at 37°C) for 12 hours, medium was replaced by fresh medium, and 48 hour later, luminescence was measured as described above. The sample ED_50_ (median effective dose) was calculated using the Reed-Muench method^4^.

**ELISA**

96-well ELISA plates were coated with RBD (Sino Bio. 40592-V08B), RBD (L452R) (Sino Bio. 40592-V08H28), S variant D614G (Sino Bio. 40591-V08H3), or other S variants (W152C, L452R, D614G) (Sino Bio. 40591-V08H17). Plates were blocked with BSA and washed. Serum samples were diluted starting from 1:2000 and performed with 1:2 serial dilutions using dilution buffer. Diluted samples were added to the corresponding wells and incubated for 1 hour at 37°C, followed by washing. Antibodies were detected with Goat anti-Monkey IgG H&L (Alkaline Phosphatase) or Goat anti-Monkey IgG H&L (Alkaline Phosphatase) where appropriate and diluted 1:1000 for a 30 min incubation at RT. After washing, Alkaline phosphatase yellow (pNPP) liquid substrate (Sigma, P7998-100ML) was added to each well and incubated for 15-20 min before reaction was stopped using 3M NaOH. Optical density was measured at 450 nm.

**RNA extraction and real-time quantitative PCR**

Total RNA was purified using a RNA mini kit (Qiagen) according to the instruction, and then cDNA generated by reverse transcription using the HiScript® III All-in-one RT SuperMix Perfect for qPCR (Vazyme). Real-time quantitative PCR reactions were set up with the FS Universal SYBR Green Master (Roche) and carried out in an iCyclerMyiQ2 Detection System (BIO-RAD). Primers used for qPCR were listed in Supplementary Table 2.

**Quantification and statistical analysis**

GraphPad Prism 8 was used for plotting and statistical analysis; the values were expressed as means ±SEM. An unpaired Student’s t test was used and p-value of less than 0.05 was considered to be significant. * P<0.05, ** P<0.01, *** P<0.005, **** P<0.001.

**Figure S1. Illustration of CAL.20C Spike-related mutant sites**


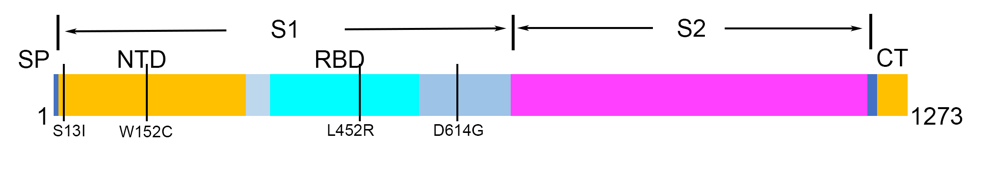


**Figure S2. High ACE2 and TMPRSS2 expression levels in 293T-ACE2-TMPRSS2 cell line**

**Figure S3. An overview of the L452R sites illustrated based on the solved complex structure of SARS-CoV-2 RBD bound to ACE2 (PDB code: 6LZG)**


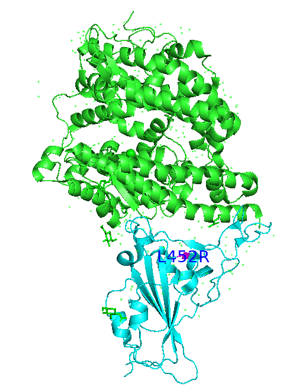


ACE2 was labeled as Green, RBD was labeled as Cyan, L452R site was labeled as Magenta.

**Supplementary Table 1. Primers used for generating mutant S variants plasmids**

| S-S13I-F | CCTCTGGTCTCCATCCAGTGCGTCAATCTGACAACTCGG |
| --- | --- |
| S-S13I-R | ATTGACGCACTGGATGGAGACCAGAGGCAGCAGGACCAG |
| S-W152C-F | AACAATAAGAGCCGGATGGAGTCCGAGTTTAGAGTGT |
| S-W152C-R | CGGACTCCATCCGGCTCTTATTGTTCTTGTGATAGTA |
| S-L452R-F | ACTACAATTATCGGTACCGGCTGTTTAGAAAGAGCAA |
| S-L452R-R | AACAGCCGGTACCGATAATTGTAGTTGCCGCCCACTT |
| S-D614G-F | TGCTGTATCAGGGCGTGAATTGTACCGAGGTGCCCGT |
| S-D614G-F | GTACAATTCACGCCCTGATACAGCACGGCCACCTGGT |

**Supplementary Table 2. Primers used for qPCR**

| hGAPDH-qPCR-F | GTCTCCTCTGACTTCAACAGCG |
| --- | --- |
| hGAPDH-qPCR-R | ACCACCCTGTTGCTGTAGCCAA |
| hACE2-qPCR-F | TGATGCTTTCCGTCTGAATGA |
| hACE2-qPCR-R | CACTCCCATCACAACTCCAA |
| hTMPRSS2-qPCR-F | CATGGCATTGGACGGCATTT |
| hTMPRSS2-qPCR-R | TTGTTCTTGGTCTTGGAGTCATA |

1. Zhang, K., Tong, W., Wang, X. & Lau, J. Y.-N. Estimated prevalence and viral transmissibility in subjects with asymptomatic SARS-CoV-2 infections in Wuhan, China. *Precision Clinical Medicine* **3**, 301–305 (2020).

2. Xu, X. *et al.* Seroprevalence of immunoglobulin M and G antibodies against SARS-CoV-2 in China. *Nature Medicine* **26**, 1193–1195 (2020).

3. Yang, J. *et al.* A vaccine targeting the RBD of the S protein of SARS-CoV-2 induces protective immunity. *Nature* **586**, 572–577 (2020).

4. Nie, J. *et al.* Quantification of SARS-CoV-2 neutralizing antibody by a pseudotyped virus-based assay. *Nature Protocols* **15**, 3699–3715 (2020).
